# Gut Microbiota-Based Immunotherapy: Engineered *Escherichia coli* Nissle 1917 for Oral Delivery of Glypican-1 in Pancreatic Cancer

**DOI:** 10.3390/medicina61040633

**Published:** 2025-03-30

**Authors:** Idris Vruzhaj, Marta Gambirasi, Davide Busato, Aurora Giacomin, Giuseppe Toffoli, Amin Safa

**Affiliations:** 1 Experimental and Clinical Pharmacology Unit, Centro di Riferimento Oncologico di Aviano (CRO) IRCCS, 33081 Aviano, Italydavide.busato@cro.it (D.B.); aurora.giacomin@cro.it (A.G.); 2 Department of Life Sciences, University of Trieste, 34127 Trieste, Italy; 3 Doctoral School in Pharmacological Sciences, University of Padua, 35122 Padova, Italy; 4 Institute of Research and Development, Duy Tan University, Da Nang 550000, Vietnam

**Keywords:** oral vaccine delivery, Glypican-1, immunotherapy, *Escherichia coli* Nissle 1917, pancreatic cancer vaccines

## Abstract

*Background and Objectives*: The administration of oral vaccines offers a potential strategy for cancer immunotherapy; yet, the development of effective platforms continues to pose a difficulty. This study examines *Escherichia coli* Nissle 1917 (EcN) as a microbial vector for the precise delivery of Glypican-1 (GPC1), a tumor-associated antigen significantly overexpressed in pancreatic ductal adenocarcinoma (PDAC).To evaluate the effectiveness of EcN as a vector for the delivery of GPC1 and assess its potential as an oral vaccination platform for cancer immunotherapy. *Materials and Methods*: EcN was genetically modified to produce a GPC1-flagellin fusion protein (GPC1-FL) to augment antigen immunogenicity. The expression and stability of GPC1 were confirmed in modified PANC02 cells using Western blot and flow cytometry, indicating that GPC1 expression did not influence tumor cell growth. A mouse model was employed to test immunogenicity post-oral delivery, measuring systemic IgG, IL-10, IL-2, and IFN-γ levels to indicate immune activation. *Results*: Oral immunization with EcN GPC1-FL elicited a robust systemic immune response, demonstrated by markedly increased levels of IgG and IL-10. IL-2 and IFN-γ concentrations were elevated in vaccinated mice relative to controls; however, the differences lacked statistical significance. Western blot examination of fecal samples verified consistent antigen expression in the gastrointestinal tract, indicating effective bacterial colonization and antigen retention. No detrimental impacts were noted, hence substantiating the safety of this methodology. *Conclusions*: These findings confirm EcN as a feasible and patient-friendly oral vaccination platform for cancer immunotherapy. The effective production of GPC1 in tumor cells, along with continuous antigen delivery and immune activation, underscores the promise of this approach for PDAC and other cancers. This study promotes microbial-based antigen delivery as a scalable, non-invasive substitute for traditional vaccine platforms.

## 1. Introduction

Pancreatic ductal adenocarcinoma (PDAC) is a highly aggressive and difficult-to-treat type of cancer. It has a very poor five-year survival rate and is commonly resistant to standard treatments. PDAC, which stands for pancreatic ductal adenocarcinoma, is the third most common cause of cancer-related deaths in the United States. In 2023, there were more than 62,200 new cases of PDAC diagnosed and it resulted in almost 48,800 fatalities [1,2]. The existing treatment options, such as surgical removal, which is the only approach with sufficient, but still temporary, efficiency, are often only suitable for a small group of people when they are diagnosed. For most people, these therapies have limited success, highlighting the need for innovative and effective treatment approaches [3,4]. Despite extensive research efforts, both systemic and molecularly targeted medicines have persistently failed to improve survival rates. This highlights the need to explore new approaches [5,6,7]. Immunotherapy has emerged as a feasible alternative [8,9], utilizing the specificity and adaptability of the immune system to precisely target PDAC. This technique aims to activate cytotoxic T lymphocytes (CTLs) that can recognize and destroy cells expressing tumor-associated antigens (TAAs) [10]. TAAs, such as Glypican-1 (GPC1) and the melanocyte differentiation antigen glycoprotein (gp100), are attractive targets due to their increased expression in tumor cells compared to normal cells. This could potentially enhance the specificity of immune responses towards tumors [11,12,13,14,15,16,17,18,19]. GPC1, a crucial heparan sulfate proteoglycan involved in key signaling pathways that promote tumor growth and spread, has generated significant attention. Its prominent expression in PDAC makes it an ideal target for immunotherapeutic efforts. Recent studies have confirmed its ability to elicit significant immunological responses in preclinical settings [18,20,21,22,23,24].

The unique feature of our work consists of utilizing genetically engineered *Escherichia coli* Nissle 1917 (EcN), a probiotic strain known for its powerful immunomodulatory qualities, to effectively transport these antigens. Historically used in the field of biotechnology, EcN has been modified to produce GPC1, thus shifting conventional vaccine delivery from invasive techniques to oral administration. Not only does this simplify the treatment plan, but it also enhances patient compliance and activates the body’s mucosal immune response, which is crucial for tackling gastrointestinal features of malignancies such as PDAC [25,26,27,28,29,30,31,32,33,34,35].

This work focuses on the creation of a reliable PANC02 cell line that produces GPC1. This cell line is used to mimic the tumor environment and allow for a thorough evaluation of the immune response. This novel strategy takes advantage of the non-invasive delivery route and the inherent immunomodulatory features of EcN to enhance the effectiveness of microbial vectors in immunotherapy.

The results of our study confirm that this approach is safe, as evidenced by the absence of negative side effects in animal models, and it induces a significant enhancement in immune system response. This proof-of-concept highlights the possibility of using EcN as a carrier for TAA delivery. It also paves the way for further study to improve and fully utilize the benefits of this promising approach in fighting pancreatic cancer and maybe other types of cancer.

## 2. Materials and Methods

### 2.1. Microbial Strains and Culture Media

The bacterial strain used in this study was the probiotic *Escherichia coli* Nissle 1917 (EcN), which was obtained from CADIgroup in Rome, Italy.

Preparation of Culture Media: Bacterial cultures were grown in Luria–Bertani (LB) medium, which consisted of 0.5% yeast extract, 1% tryptone, and 1% sodium chloride by weight. The solid medium formulations were prepared by using a 2% concentration of agar. The components were dissolved in distilled water and then sterilized by autoclaving at a temperature of 120 °C for a duration of 20 min, using a PBI Sistematic III autoclave (Gemini, Apeldoorn, The Netherlands).

Culturing Conditions: In order to facilitate the growth of genetically engineered bacteria, the medium was enriched with 100 µg/mL of ampicillin. The liquid cultures were placed in a 222DS Benchtop Shaking Incubator (Labnet International, Edison, NJ, USA) and kept at a constant temperature of 37 °C, with the agitation speed set at 250 rpm. The solid cultures were kept at a constant temperature in a Heraeus incubator (Gemini, Apeldoorn, The Netherlands).

### 2.2. Cell Line and Culture Conditions

The PANC02 cell line, which is commonly used as a model for mouse pancreatic ductal adenocarcinoma (PDAC), was created by chemically inducing it with 3-methylcholanthrene (3-MCA). The origin of this cell line may be traced back to CLS Cell Lines Service GmbH, The Germany.

The PANC02 cells were grown in RPMI-1640 medium with 10% fetal bovine serum (FBS), 100 U/mL penicillin, 100 µg/mL streptomycin, 1 mM nonessential amino acids, and 1 mM sodium pyruvate. The cultivation took place in a humidified incubator, where the environment was maintained at 95% air and 5% CO_2_, and the temperature was kept constant at 37 °C.

### 2.3. Transfection and Confirmation of GPC1 Expression in PANC02 Cells

Transfection Procedure: The Amaxa™ Cell Line Nucleofector™ Kit L (Lonza) The Italy, a highly efficient electroporation technique, was used to achieve the transfection of PANC02 cells. This enables the accurate delivery of genetic material into mammalian cells. The cells were transfected with a plasmid harboring the coding sequence of the human GPC1 gene combined with a puromycin resistance gene using electroporation. After electroporation, the cells were cultured in a medium containing 3 μg/mL puromycin to specifically choose cells that had effectively incorporated the GPC1 plasmid. This selection process promoted the enhancement of a group of cells that expressed the GPC1 gene.

Validation of GPC1 Expression: In order to verify the presence of the GPC1 antigen on the cell surface, cells that had been genetically modified were treated with a fixative and then stained using a specialized antibody that targets GPC1. Flow cytometry was used to evaluate the presence of GPC1 on the cell membrane. Furthermore, Western blot analysis was employed to confirm the existence of the protein, guaranteeing a thorough validation of GPC1 expression and functionality in the transformed PANC02 cell line.

Cell growth impact assessment: In addition to validating the expression of GPC1, we assessed the cell doubling time of both the transfected and wild-type PANC02 cell lines to investigate any impact of GPC1 expression on cellular proliferation. When the cell culture reached around 80% confluence, the cells were separated using trypsin and then quantified using a hemocytometer. The cell doubling time was determined using the conventional formula:

$$Doubling\;time=\frac{Duration\times In(2)}{In(final\;concentration÷initial\;concentration)}$$

### 2.4. Construction and Validation of Expression Vectors

VectorBuilder’s services were utilized to engineer the expression vector pVB2gcp1, which was specifically built for expressing glypican-1 (GPC1) in *Escherichia coli* Nissle. The vector has a constitutive promoter obtained from the glyceraldehyde-3-phosphate dehydrogenase (GAP) gene, which guarantees consistent levels of expression. VectorBuilder employed their patented codon optimization approach to improve the expression of the coding sequence for human GPC1 in *E. coli*. Moreover, the inclusion of a GAP terminator guarantees a conclusive conclusion to the transcriptional unit. The pUC19 plasmid contains an origin of replication, which allows it to replicate within bacterial hosts.

Verification of Vector: The integrity and proper assembly of the pVB2gcp1 plasmid were verified via restriction enzyme analysis. The BamHI and XhoI enzymes, obtained from New England Biolabs (NEB) in Ipswich (MA, USA), selectively targeted particular restriction sites adjacent to the GPC1 gene. The digestion patterns obtained confirmed the precise integration of the glypican-1 gene into the vector.

Alternative Vector Engineering: In addition to using the commercially available pVB2gcp1 vector, classic molecular cloning procedures were used to create alternative expression vectors. The GPC1 gene was identified by a “cut-and-sew” technique, which employed the deliberate application of restriction enzymes and T4 DNA ligase (NEB). Subsequently, this gene was included in other pVB2 plasmid structures, hence expanding the range of options available for conducting comparative expression investigations. This versatile technique enables a comprehensive assessment of GPC1 expression kinetics across various vector combinations.

### 2.5. Western Blot Analysis

Sample Preparation: Bacterial colonies that were genetically modified to carry the glypican-1 expression vector were chosen from agar plates that contained ampicillin. The cells were grown in 20 mL of LB media with an additional 100 µg/mL of ampicillin, maintained at a constant temperature of 37 °C. The culture, driven by a constantly active promoter, grew fast without requiring an inducer. The bacterial cells were collected by subjecting them to centrifugal force at a temperature of 4 °C using an Eppendorf 5810R centrifuge (Eppendorf, Hamburg, Germany). After centrifugation, the liquid above the sediment was removed, and the sediment containing the cells was mixed with 3 mL of lysis buffer. The lysis buffer consisted of 25 mM Hepes, 0.2 M NaCl, 5% glycerol, a protease inhibitor cocktail without EDTA, 1 µg/mL DNAse, and 0.1% Triton X-100.

The PANC02 cell line was prepared by dissociating the cells using trypsin and then collecting the resulting cell pellet using centrifugation. The cells were suspended in 30 µL of RIPA solution containing 200 mM NaF, 40 mM Na_3_VO_4_, a 25× EDTA-free cocktail, 0.05 M EDTA, and normal RIPA buffer for lysis. The mixture was placed on ice and incubated for 20 min. It was then subjected to centrifugation at a speed of 12,000 revolutions per minute for 20 min at a temperature of 4 °C. After centrifugation, the liquid portion containing the broken-down proteins was collected.

Cell Disruption and Protein Extraction for Bacterial Samples: The bacterial cells that were suspended again were exposed to sonication for a duration of 20 s at a power level of 40% while being kept cold on ice. This was performed using a Bandelin Sonopuls 3200 device equipped with MS72 sonotrodes (manufactured by Bandelin, Berlin, Germany). This approach was designed to effectively break down the cells. The protein concentration of both bacterial and PANC02 cell samples was measured using a Bradford assay with a Nanodrop 2000c Spectrophotometer (Thermo Fisher Scientific, Waltham, MA, USA).

SDS-PAGE and Protein Transfer: An accurately measured 20 µg of protein from each sample type was placed onto a Mini-PROTEAN TGX precast gel (Bio-Rad, Hercules, CA, USA). Subsequently, proteins were transferred on a nitrocellulose membrane (GE Healthcare, Milan, Italy) using traditional wet transfer techniques.

Immunoblotting: The membrane was obstructed with a 5% non-fat milk solution in TBST for a duration of one hour at room temperature. Afterwards, the sample was kept at a temperature of 4 °C overnight and treated with a primary anti-GPC1 antibody, which was diluted to a concentration of 1:1000 (catalog # PA5-28055, Thermo Fisher Scientific). Following three washes in TBST, the membrane was exposed to an HRP-conjugated secondary antibody, which was diluted at a ratio of 1:5000 (catalog # ab6721, Abcam, Cambridge, UK), and incubated for one hour at room temperature.

Protein bands were detected by employing enhanced chemiluminescence (ECL) reagents and recorded using a ChemiDoc XRS+ digital imaging system with Image Lab 6.1 software (Bio-Rad, Hercules, CA, USA). The meticulous methodology employed ensured precise detection and examination of GPC1 protein expression in both genetically modified bacterial cells and PANC02 cell lines.

### 2.6. Analysis of GPC1 Expression on PANC02 Cells Using Flow Cytometry

Flow cytometry was used to quantitatively examine the presence of Glypican-1 (GPC1) on the surface of PANC02 cells. This analysis was primarily conducted to aid in the accurate selection of positive cells. At first, some 500,000 cells were removed from growth plates and treated with Dulbecco’s Phosphate-Buffered Saline (DPBS) containing 1% Bovine Serum Albumin (BSA) to prevent further reactions. The cells were then cultured with a Glypican-1 polyclonal antibody (PA5-28055, Thermo Fisher Scientific, 0.66 mg/mL) that was diluted at a ratio of 1:100 in PBS solution containing 2% BSA. The incubation was conducted for a duration of one hour at ambient temperature with constant agitation to ensure thorough antibody exposure. After the incubation period, the cells were rinsed two times with the blocking solution in order to remove any antibodies that were not bound.

After the initial binding of the antibody, the cells were then exposed to a secondary antibody called F(ab’)2 Goat anti-Rabbit IgG-F(ab’)2 Fragment, which was cross-adsorbed and conjugated with DyLight^®^ 488. This specific secondary antibody is listed as Catalog A120-212D2 and is manufactured by Bethyl Laboratories Inc, The Germany. The incubation was conducted for a duration of one hour at ambient temperature with continuous stirring. Afterward, the cells were rinsed two additional times with the blocking solution to eliminate any remaining surplus secondary antibodies.

During the final phase of data acquisition, the cells were mixed with 350 µL of the blocking solution and analyzed using a BD FACSCanto™ II Cell Analyzer (Becton, Dickinson and Company, Franklin Lakes, NJ, USA). The gathered data were further processed and analyzed using BD FACSDiva Software, version 9.0. The meticulous and systematic methodology employed guaranteed the accurate measurement of GPC1 expression, therefore yielding a comprehensive comprehension of the dynamics of antigen presentation on the surface of PANC02 cells.

### 2.7. Protocol for Immunization and Sampling to Evaluate Immune Responses

Animal Model Description: The study employed a total of fifteen C57BL/6 N mice, which were between 6 and 8 weeks old. These mice were obtained from Charles River Laboratories International, Inc. The mice were randomly allocated into three groups of five for oral vaccination experiments. The initial group, referred to as the EcN GPC1-FL group, received a dose of genetically modified EcN bacteria that expressed Glypican-1 together with Flagellin. The second group, known as the EcN WT group, was administered the wild-type strain of *Escherichia coli* Nissle 1917. The third group, referred to as the PBS group, received PBS as a control substance.

**Figure 1:**
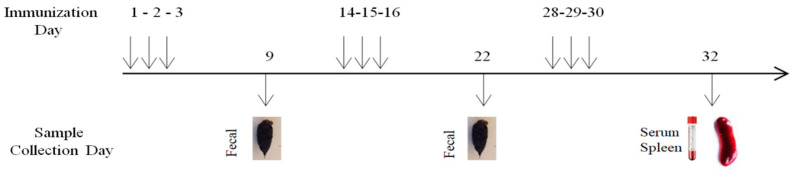
Visualizing immunization: a schematic overview.

Vaccination Procedure: The vaccination protocol consisted of delivering 1.0 × 10^9^ colony-forming units in a volume of 100 µL. The available vaccine choices were EcN GPC1-FL, EcN WT, or a 100 µL PBS control solution. The vaccinations were administered on days 1–3, 14–16, and 28–30 using both the gavage method and mixing the vaccine with food to ensure consistent ingestion over each three-day immunization interval (Figure 1). During periods other than these specified time frames, mice were allowed to freely consume regular food and water ad libitum.

Collection of Samples and Ethical Considerations: On the 32nd day, all mice were euthanized in a humane manner utilizing CO_2_ hypoxia. Their spleens were then extracted under sterile circumstances for further analysis in vitro. The experimental design underwent thorough evaluation and received approval from the institutional ethics and animal committee of the CRO. This ensures that the research adheres to the highest standards of animal welfare and ethical conduct.

### 2.8. Isolation and Protein Analysis of EcN Expressing GPC1-FL from Fecal Samples

Fecal Sample Collection and Analysis: In order to confirm the successful establishment and continued survival of orally delivered EcN GPC1-FL, fecal samples were systematically obtained from the specified groups both before and after vaccination, as shown in Figure 1. Pre-immunization samples were collected as a reference point for comparison. The fecal material that was gathered was mixed well in Luria–Bertani (LB) broth to produce consistent suspensions. Subsequently, these suspensions were spread onto LB agar plates containing ampicillin in order to preferentially grow ampicillin-resistant strains of EcN. Following incubation, bacterial colonies were isolated and cultured in liquid LB broth at a temperature of 37 °C for a duration of 24 h to ensure their vigorous growth. This procedure was developed to assess the durability of EcN and its antigen expression in the gastrointestinal tract of mice.

Protein Isolation and Western Blot Analysis: In order to verify the existence of the GPC1-FL antigen in the bacteria obtained from fecal samples, proteins were isolated from the cultivated samples and examined using Western blotting techniques. This method not only showed that the genetically modified bacteria were present in the mice’s intestines but also confirmed that the functional antigen was continuously produced when it was given to them. The combination of culture and protein analysis in this two-step procedure yielded strong evidence of both the presence and functional effectiveness of the altered EcN strains in the host system.

### 2.9. ELISA Assay for Quantifying IL-2, IFN-γ, and IL-10 in Splenocytes and Serum

The procedure for cell preparation and cultivation entailed collecting splenocytes from the spleens of immunized mice, filtering to exclude extraneous material, and subsequently performing hemolysis to remove red blood cells. The cells were then suspended in RPMI-1640 media, enriched with 10% fetal bovine serum (FBS), 10 mM HEPES, 1 mM nonessential amino acids, 1 mM sodium pyruvate, 50 µM 2-mercaptoethanol, 100 IU/mL penicillin, and 100 µg/mL streptomycin. Splenocytes were inoculated into 96-well microplates at a density of 4 × 10^5^ cells per well, with 200 µL of media supplemented in each well.

Splenocyte Activation: Splenocytes were activated through exposure to mitomycin C-treated PANC02-GPC1 cells or control PANC02 cells at a density of 4 × 10^4^ cells per well. The cultures were incubated at 37 °C with 5% CO_2_ for 72 h, after which the supernatants were harvested for cytokine analysis.

Sample Preservation and Analysis: After the stimulation phase, the culture medium was collected and preserved at −80 °C until cytokine measurement. Concurrently, blood samples were obtained from the mice and stored at −80 °C for the assessment of interleukin-10 (IL-10) concentrations.

Cytokine levels were quantified using ELISA kits specific for interleukin-2 (IL-2, BMS601), interferon-gamma (IFN-γ, KMC4021), and interleukin-10 (IL-10, BMS614TEN), supplied by Thermo Fisher Scientific, Inc. The concentrations of IL-2 and IFN-γ in the culture media, along with IL-10 in serum, were assessed according to the manufacturer’s instructions. This method guaranteed a precise evaluation of the immunological response elicited by the vaccination protocol.

### 2.10. ELISA for the Detection of Specific Anti-GPC1 Antibodies

The Nunc Thermo Fisher 96-well plates were coated with 800 ng of eukaryotic glypican-1 protein (LS-G40291/145511) using carbonate-bicarbonate buffer (pH 9.6) and left to incubate overnight at 4 °C. The plates were rinsed thrice with PBS solution containing 0.05% Tween-20 (PBS-T) and then treated with a 5% non-fat milk solution in PBS-T for two hours at room temperature to reduce any non-specific binding. The serum was acquired through the process of centrifuging blood samples from mice. It was then diluted in PBS with 1% Tween and introduced to the wells that had been coated. The mixture was left to incubate at room temperature for a duration of two hours. After that, the wells were rinsed three times with PBS-T and then treated with an HRP-conjugated secondary antibody that specifically targets mouse IgG (1:1000, catalog # AMab6721-1mg) for a duration of one hour. Following repeated washes, TMB substrate was introduced to each well, and the colorimetric reaction was allowed to proceed for a duration of 10–20 min in the absence of light. The reaction was stopped by adding 1N H_2_SO_4_, and the absorbance was quantified at 450 nm using a microplate reader.

### 2.11. Statistical Analysis

Statistical analyses were conducted using SPSS software, version 16.0, specifically version 16. The comparison of several groups was conducted using a one-way Analysis of Variance (ANOVA), with further utilization of Tukey’s post hoc test for comprehensive pairwise comparisons. In order to make specific comparisons between two separate groups, a *t*-test was used to identify any significant differences. The statistical significance was established with a *p*-value of less than 0.05, demonstrating significant differences between the experimental and control groups.

## 3. Results

### 3.1. Enhanced Expression and Immunogenic Potential of Glypican-1-Flagellin Fusion Protein in Engineered Escherichia coli Nissle 1917

This study focuses on the development and analysis of a sophisticated expression vector, pVB2gpc1, designed to enhance the production of GPC1 in *Escherichia coli* Nissle 1917 (EcN). The vector includes a GAP constitutive promoter and a GAP terminator to guarantee constant and efficient transcription. Additionally, the cDNA sequence of human Glypican-1 was altered to improve the utilization of codons and increase bacterial production. This involved adding a 6xHis tag at the C-terminal end to simplify the purification process. The vector is equipped with a pUC19 origin of replication and an ampicillin resistance gene, facilitating fast replication and selective growth.

**Figure 2:**
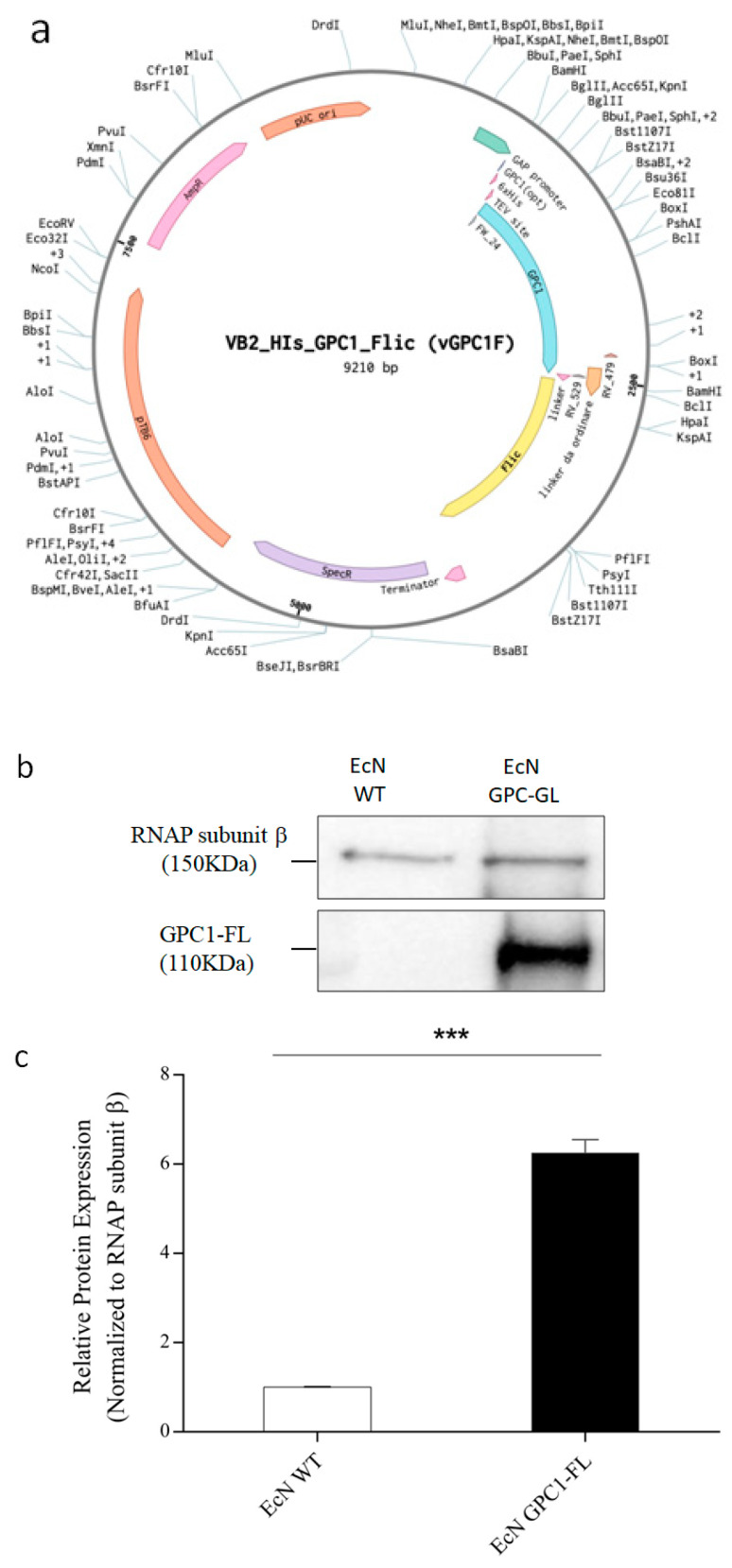
Development and Verification of GPC1 and GPC1-Flagellin Expression in *Escherichia coli* Nissle. (**a**): Diagram Description: The diagram depicts the composition of the pVB2gpc1 vector, which is designed for the production of GPC1 and the GPC1-Flagellin fusion protein. The diagram highlights key genetic components, such as the GAP promoter for consistent gene expression, optimized GPC1 cDNA for increased production in bacteria, integration of the FliC gene from Salmonella Typhimurium to enhance immunogenicity, and transcriptional terminators to mark the end of the transcription unit. (**b**): The Western blot (**b**) and the corresponding quantitative graph (**c**) present protein samples from EcN-WT and EcN GPC-FL. The RNA polymerase subunit β (RNAP subunit β, 150 kDa) functioned as a loading control to verify uniform protein loading across lanes. The GPC1-Flagellin (GPC1-FL, 110 kDa), was distinctly expressed alone in the transfected EcN (EcN GPC1-FL), signifying effective transfection and expression. Protein expression was measured using ImageJ software version 1.42, adjusted to RNAP subunit β, and the results indicated that expression levels of GPC1-FL were considerably elevated in the transfected strain relative to the wild type (*** *p* < 0.001). The experiment was conducted thrice, highlighting the reliable and effective augmentation of GPC1-FL transfected EcN.

In order to enhance the capabilities of the vector, pVB2gpc1 underwent alterations utilizing SLIC cloning techniques. These modifications involved the introduction of the flagellin gene (FliC) from Salmonella Typhimurium to the C-terminal of GPC1. The incorporation of the linker sequence, consisting of four repetitions of Glycine-Glycine-Glycine-Serine (GGGS), was deliberately designed to guarantee proper folding and autonomous activity of the merged proteins (see Figure 2a). This alteration exploits the powerful immunostimulatory capabilities of flagellin, which is known for its ability to activate TLR-5, thus increasing the immunogenicity of GPC1.

After introducing this genetic construct into EcN by electroporation, the presence of the resulting proteins was verified by performing a Western blot analysis. The examination of the GPC1-Flagellin fusion revealed a band with a molecular weight of approximately 110 kDa, which aligns with the anticipated size of the combined protein. It is worth mentioning that the occasional existence of a smaller band indicates the possibility of partial fusion or separate expression of GPC1 (refer to Figure 2b and Supplementary Figure S1 for the original Western blot images).

### 3.2. Expression of GPC1 and Growth Dynamics in Engineered PANC02 Cells

**Figure 3:**
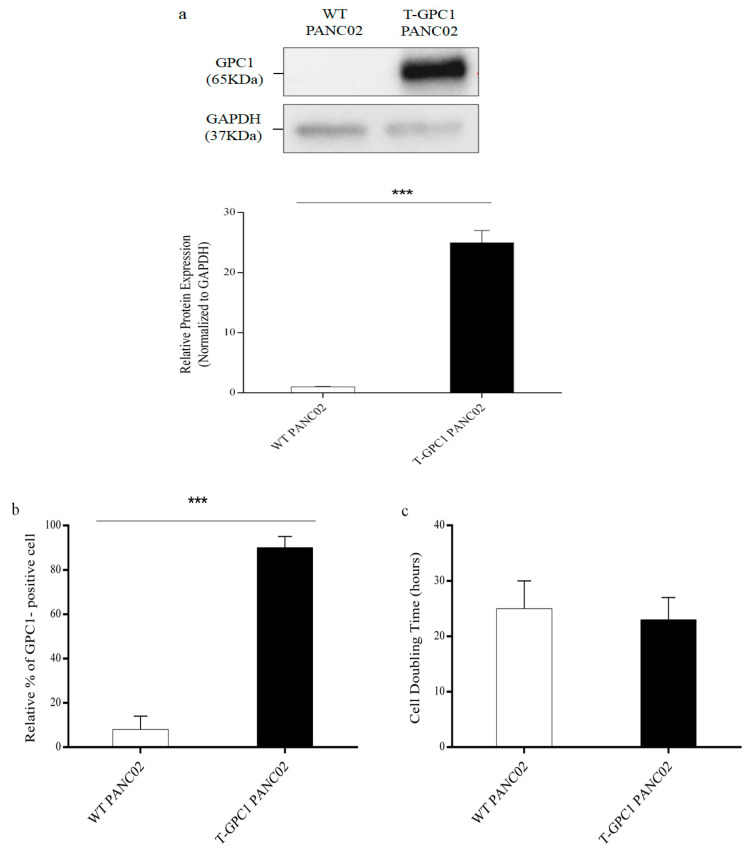
An analysis of GPC1 protein expression and proliferation in engineered PANC02 cells. (**a**): GPC1 protein expression in PANC02 cells. The picture illustrates a Western blot study contrasting GPC1 protein expression in wild-type (WT) and genetically modified PANC02 cells (T-GPC1 PANC02). The upper panel indicates the presence of GPC1 (65 kDa) solely in the transfected cells, while it is lacking in WT cells. GAPDH (37 kDa) serves as a loading control, guaranteeing uniform protein loading among the samples. The lower panel quantifies relative protein expression, normalized to GAPDH, demonstrating a considerably elevated expression of GPC1 in T-GPC1 PANC02 cells compared to WT, as denoted by the triple asterisks (*** *p* < 0.001). (**b**): Flow cytometry for GPC1 surface expression: The flow cytometry data show a notable elevation in the expression of GPC1 on the surface of T-GPC1 PANC02 cells as compared to the WT group. The increased expression on the surface of the cell provides evidence for the functional existence of GPC1 as a result of the transfection. (**c**): Cell doubling time analysis: This graph presents a comparison of the cell doubling durations between WT and T-GPC1 PANC02 cells. The data indicates that the introduction and subsequent activation of GPC1 through transfection does not have a detrimental effect on the growth rate of the cells, suggesting that the normal course of the cell cycle is maintained. Statistical analysis: The statistical significance was evaluated using an unpaired *t*-test. Significant differences in GPC1 surface expression between the wild-type (WT) and T-GPC1 PANC02 cells were seen at a *p*-value below < 0.001.

The work successfully showed the presence of GPC1 in the PANC02 pancreatic cancer cell line and assessed its impact on cellular proliferation. The presence of GPC1 in transfected PANC02 cells (T-GPC1 PANC02) was confirmed by Western blot analysis, as evidenced by a distinct protein band representing GPC1 (65 kDa). In contrast, the wild-type (WT) PANC02 sample showed no expression of GPC1, underscoring the accuracy and effectiveness of the transfection procedure(Figure 3a and Supplementary Figure S2). Flow cytometry analysis (Figure 3b and Supplementary Figure S3) confirmed that T-GPC1 PANC02 cells exhibited a significant increase in surface expression of GPC1 compared to the WT group. This finding supports the presence and functional expression of the antigen on the cells.

In addition, an examination of cell doubling times (Figure 3c) revealed no significant difference in proliferation rates between the WT PANC02 and T-GPC1 PANC02 groups. This discovery suggests that the incorporation and manifestation of the GPC1 antigen did not have a negative effect on the growth patterns of the cells. This supports the possibility of using this genetic modification in future experiments and treatments.

### 3.3. Safety Assessment of Oral Administration of EcN GPC1-FL in Mice

**Figure 4:**
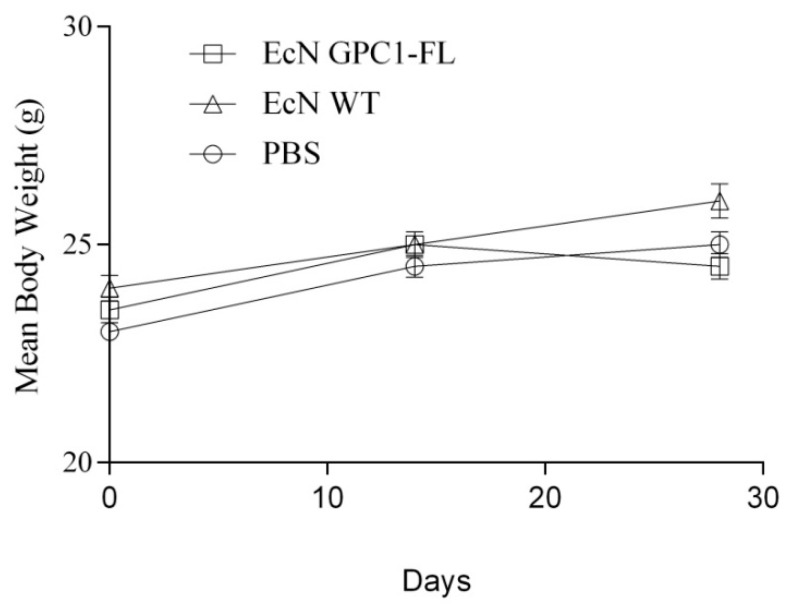
Body weight changes following oral vaccinations. This graph illustrates the weekly body weights of mice, with a sample size of 5 per group, who were administered EcN GPC1-FL, EcN WT, or PBS. Each data point represents the mean value of the group, while the error bars indicate the standard error (±SE). The mean body weight trends for all treatment groups exhibit typical growth, with no statistically significant deviations observed among the groups.

Orally administering EcN GPC1-FL to mice did not cause any significant alterations in body weight, suggesting the lack of any harmful effects. Given that GPC1 is present in normal cells and tissues [36], we thoroughly examined the possibility of self-GPC1 autoaggression after immunization. During the immunization period, the body weights of the mice were closely monitored, and no significant changes were found among the different groups (Figure 4). In addition, there were no apparent indications of toxicity, such as unkempt fur, hunched posture, or lack of energy, all of which confirm the vaccine’s safety profile.

### 3.4. Confirmation of Targeted GPC1-FL Expression in Modified Escherichia coli Nissle Following Oral Vaccination

**Figure 5:**
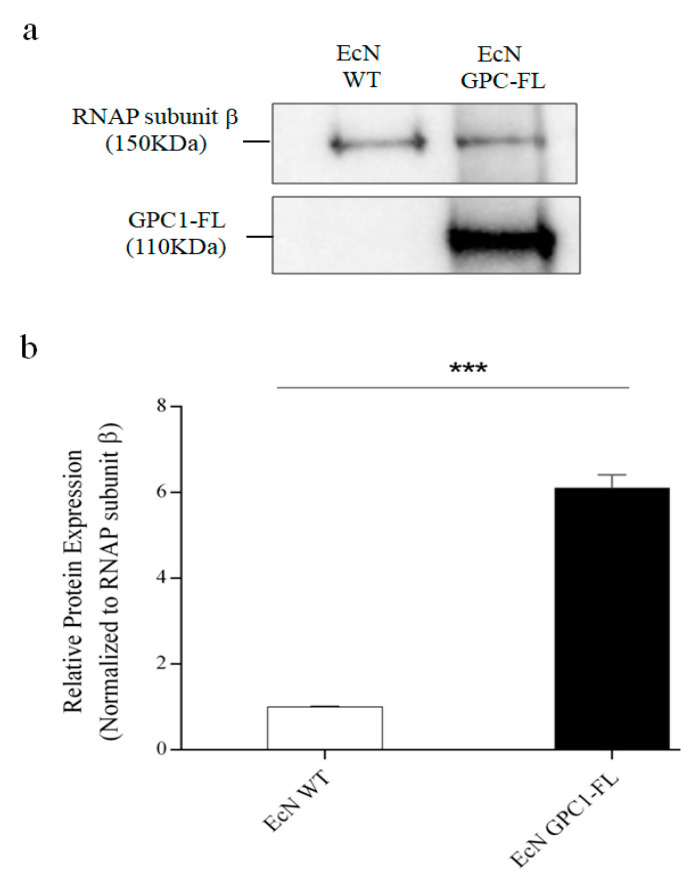
Specific expression of GPC1-FL in engineered EcN post-vaccination. The data illustrate Western blot (**a**) and densitometric analysis (**b**) of GPC1-FL in EcN obtained from mouse feces following oral immunization. Feces from three mice in each immunized group (EcN GPC1-FL and EcN WT) were utilized for Western blotting. The beta component of RNA polymerase (150 kDa) functioned as a loading control to verify uniform protein loading among the samples. The presence of GPC1-FL (110 kDa) was verified in the re-isolated EcN GPC1-FL, demonstrating persistent gene expression following immunization. Expression was measured via ImageJ and normalized to RNA polymerase β, indicating consistently elevated levels of GPC1-FL (*** *p* < 0.001).

The effectiveness of genetically engineered *Escherichia coli* Nissle (EcN) in producing the GPC1-FL protein in the gastrointestinal system was comprehensively evaluated. Fecal samples were taken after immunization and examined using Western blot. Baseline control samples were also obtained before any intervention. The Western blot results were definitive, showing that the GPC1-FL protein was only found in the fecal samples from the EcN GPC1-FL group. Neither the wild-type EcN group nor the PBS control group showed any presence of GPC1-FL in the fecal samples. This was also observed in the samples taken prior to vaccination, as shown in Figure 5 and Supplementary Figure S4. The results of this study demonstrate that GPC1-FL is expressed specifically and provide evidence that the modified bacteria successfully colonize and function in the gastrointestinal system. This highlights the potential of using this technique for targeted therapeutic interventions.

### 3.5. Elevated IgG Antibody Responses in Mice Immunized with Engineered Escherichia coli Nissle 1917 Expressing GPC1-FL

**Figure 6:**
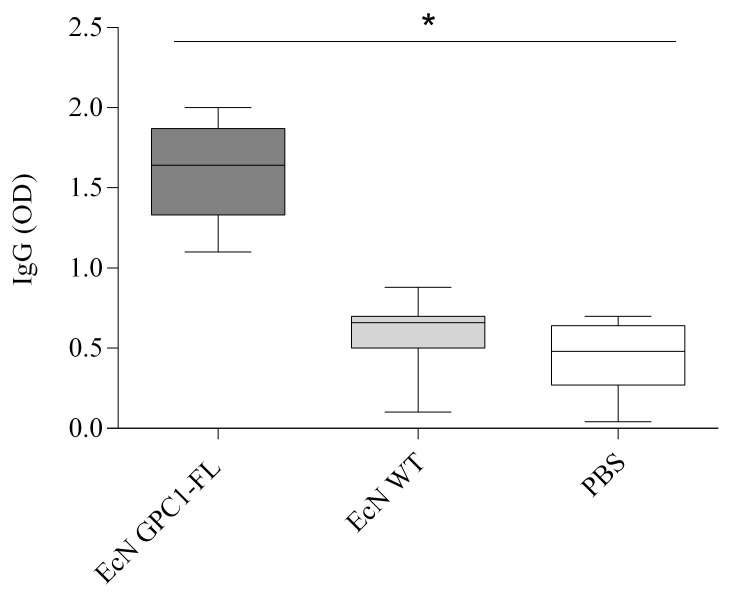
Box and whisker plot of IgG antibody levels following vaccination with EcN-GPC1-FL.

We evaluated the immune response generated by an orally administered vaccination containing genetically modified *Escherichia coli* Nissle 1917 (EcN), designed to produce the GPC1-FL protein. ELISA was used to measure the levels of IgG antibodies thirty days following vaccination. The findings revealed a notable increase in IgG levels in mice who received the EcN GPC1-FL vaccine, in comparison to those that were given the wild-type EcN vaccine and the PBS control group (as illustrated in Figure 6). This significant rise highlights the heightened ability of the GPC1-expressing EcN to stimulate an immunological response, indicating the successful presentation of antigens and subsequent activation of the immune system. The results emphasize the modified EcN’s capacity to stimulate a strong humoral immune response, making it a very intriguing option for further investigation in the field of cancer immunotherapy.

Figure 6 displays a box and whisker figure showing IgG antibody levels in mice across three experimental groups: EcN GPC1-FL, wild-type EcN (EcN WT), and PBS controls. The plot shows the distribution of IgG antibody levels, encompassing the median, interquartile range, and outliers, over all three experimental groups. The group immunized with EcN GPC1-FL demonstrated a markedly elevated IgG response relative to both the wild-type EcN and PBS cohorts, with a *p*-value inferior to * *p* < 0.05. Data points denote the IgG antibody levels (optical density, OD) assessed 30 days post-vaccination, comprising 5 mice per group from three different trials.

### 3.6. Analysis of the Immune Response After Oral Administration of EcN GPC1-FL

**Figure 7:**
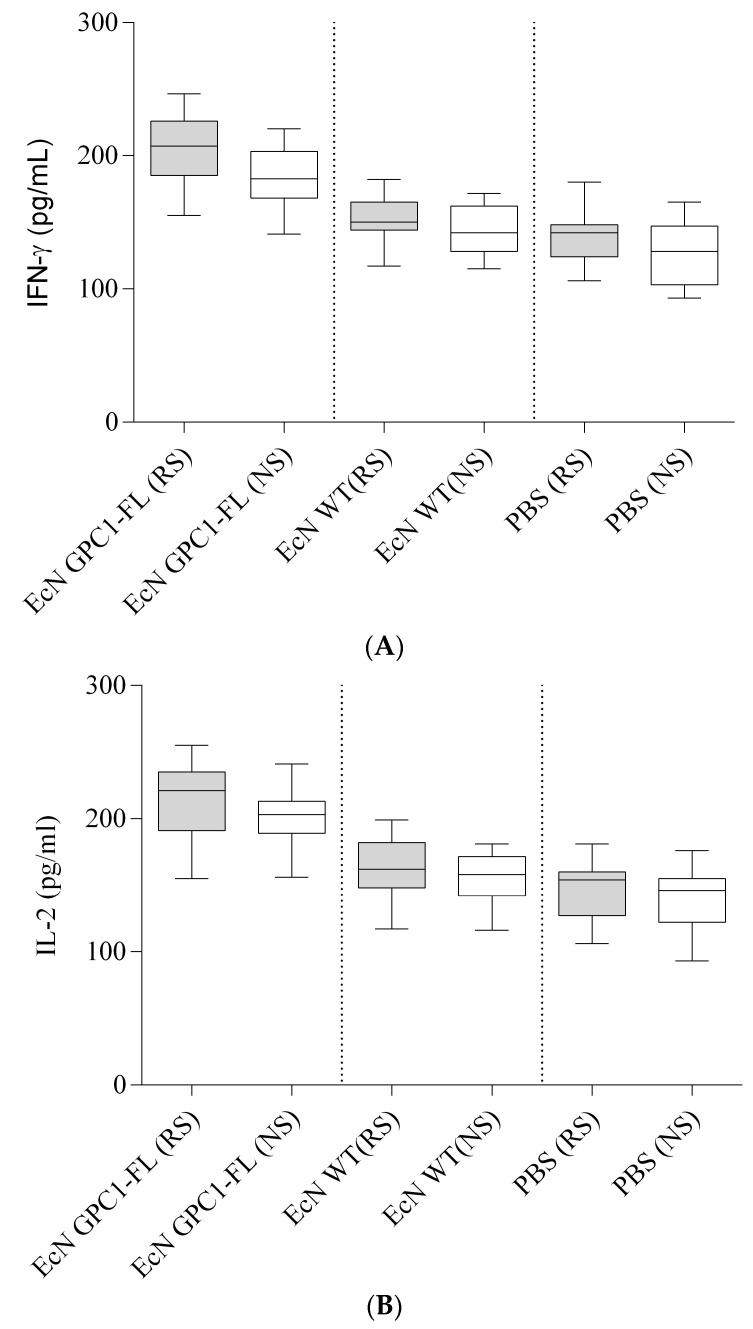
Box and whisker plots of IL-2 and IFN-γ levels post-vaccination. (**A**). IFN-γ levels: The graph displays IFN-γ concentrations in cell cultures activated by PANC02-GPC1 or PANC02, 30 days following immunization with EcN GPC1-FL, EcN WT, or PBS. The vaccinated group exhibited increased IFN-γ levels under both re-stimulated (RS) and non-stimulated (NS) conditions; however, no significant differences were noted between the groups. (**B**). IL-2 Levels: Likewise, IL-2 concentrations were elevated in the EcN GPC1-FL group relative to the other groups under both re-stimulated (RS) and non-stimulated (NS) circumstances. Nonetheless, no statistically significant differences were detected.

Following vaccine administration, we assessed the immune response by measuring the concentrations of IL-2 and IFN-γ in cell cultures stimulated with either PANC02-GPC1 or PANC02. The ELISA results for both IL-2 and IFN-γ exhibited no significant differences among the three groups. Despite the vaccinated group demonstrating elevated levels of IL-2 and IFN-γ in both stimulated and unstimulated cells, these alterations did not attain statistical significance, as depicted in Figure 7.

**Figure 8:**
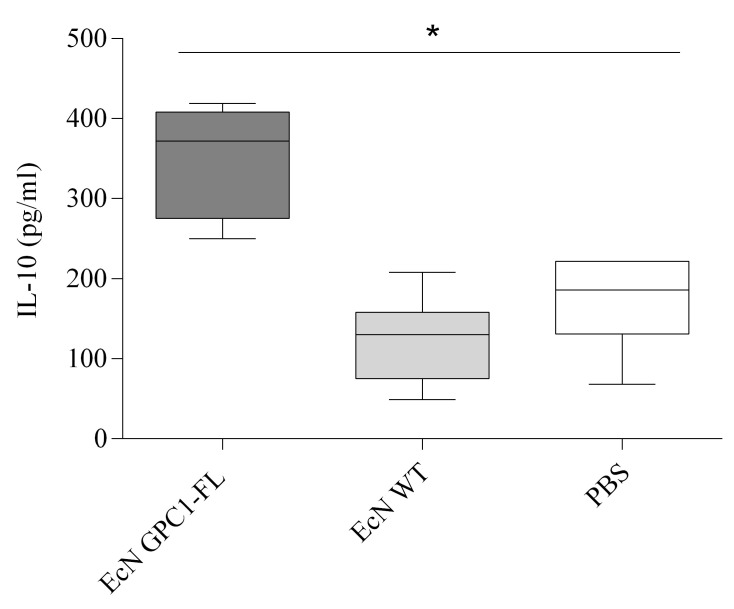
Serum IL-10 levels indicative of immune regulatory activity post-vaccination.

In addition, we assessed the immunosuppressive reaction by quantifying the levels of interleukin 10 (IL-10) in the serum of mice that were administered the vaccine. The levels of IL-10 were significantly elevated in the EcN GPC1 group compared to the other two groups. The greatest significant difference was detected between the EcN GPC1 group and the group that received only EcN WT (*p* < 0.05), as shown in Figure 8.

Figure 8 displays the IL-10 concentrations in serum samples from mice immunized with EcN GPC1-FL, EcN WT, and PBS. The EcN GPC1-FL group demonstrated markedly elevated IL-10 levels relative to both the EcN WT and PBS groups, indicating a pronounced immunoregulatory impact elicited by the modified vaccination. The box and whisker diagram shows the distribution of IL-10 levels, encompassing the median, interquartile range, and outliers, with statistical significance indicated by the asterisk (* *p* < 0.05). The data provide the average cytokine level for each group, derived from five mice per group across three different experiments.

## 4. Discussion

The study highlights the effective administration of the *Escherichia coli* Nissle 1917 (EcN) GPC1-FL vaccine by the mouth and its ability to establish itself in the intestines. We established the effectiveness of EcN, a commensal bacteria found in the human gut flora, as a safe and efficient carrier of antigens, making use of its low-affinity and well-tolerated properties. The validation process involved isolating and propagating EcN strains from stool samples. This confirmed that the strains were able to survive the severe environment of the digestive system while maintaining their genomic integrity and expressing tumor-associated antigens (TAAs) such as GPC1 [32,37,38,39,40].

In addition, our research adds to the growing body of evidence that shows EcN’s ability to transport several antigens associated with infectious and viral illnesses [35,41,42,43]. We have found that genetically modified EcN can successfully produce TAAs, which is a big breakthrough in the field of bacterial-based vaccination techniques.

The mouse model showed a substantial immunological response when orally vaccinated with GPC1-expressing *Escherichia coli* Nissle 1917. The significant rise in serum IgG levels highlights a strong humoral immune response, possibly triggered by exposure to antigens in the mucosa-associated lymphoid tissue (MALT) and subsequent activation of B cells. It is probable that these B cells undergo class switching in lymph nodes near the area before moving to the lamina propria. This process helps connect the local mucosal and systemic immune systems [44,45]. This observation is critical, as it illustrates the capacity of mucosally delivered antigens to not only initiate local immune activation but also potentiate a systemic immune response. The systemic IgG response observed may be attributed to the inherent dynamics of bacterial interaction within the immune system. Under steady-state conditions, bacterial antigens can translocate across mucosal barriers and systematically prime secondary lymphoid structures. This process, involving either bacterial particles or intact bacteria, is known to activate commensal-specific B cells, fostering a diverse and adaptable antibody repertoire predominantly comprising IgG. Such a repertoire is crucial for preventing systemic infections by enhancing immune readiness against bacterial antigens, thus transcending the localized gut immune response that predominantly induces IgA production [46].

Nevertheless, our study’s analysis of cytokine profiles uncovered a fascinating element of the immunological response. Despite the presence of a strong humoral response, as shown by increased IgG levels, there was no notable alteration in interleukin 2 (IL-2) levels, which is a crucial indicator of T cell activity. Conversely, we noticed a significant rise in interleukin 10 (IL-10) levels in the vaccinated group as compared to the control group. This pattern indicates a complex process of activation and regulation involving CD4+ T cells. It begins with the detection of antigens by T cell receptors on MHC class II molecules, followed by a second signal from B7 molecules on antigen-presenting cells [46,47]. CD4+ T cell activation did not result in a conventional inflammatory reaction, but instead led to the development of T regulatory type 1 (Tr1) cells. Tr1 cells, renowned for their immunoregulatory function and robust IL-10 secretion, indicate a prevailing mode of immune regulation rather than inflammation [47]. The cytokine response profile emphasizes the intricate nature of inducing a customized immune response by mucosal vaccinations while maintaining a delicate balance between immunogenicity and tolerance. The increased levels of IL-10 and the absence of a simultaneous inflammatory response from T cells may indicate the distinct tolerogenic conditions present in the intestinal mucosa. In this setting, immune responses are typically biased towards control in order to preserve the balance of the mucosal system [48]. The findings indicate that mucosal delivery of GPC1 introduces TAAs to the immune system but results in more regulatory than protective outcomes, suggesting potential optimization strategies for enhancing T cell activation. Moreover, in comparison to lactic acid bacteria (LAB) such as Lactococcus lactis and Bifidobacterium spp., EcN has substantial benefits as a microbial vector. LAB, however secure and efficient in generating mucosal immunity, necessitate strain-specific, inducible genetic systems and are devoid of inherent tumor-targeting abilities [49]. Koichi Kitagawa and associates revealed that Bifidobacterium longum, modified to express Wilms’ tumor 1 (WT1) protein, can elicit tumor-specific immune responses and inhibit tumor proliferation in mouse models of leukemia, prostate, and bladder cancer [27,50,51]. Bifidobacterium spp. encounter significant constraints, including oxygen sensitivity, reduced transformation efficiency, and a scarcity of well-characterized plasmid systems [52,53]. Conversely, EcN exhibits genetic stability, can be readily modified through its cryptic plasmids without the necessity of external inducers, and demonstrates resilience across many settings [35,54]. EcN exhibits inherent tumor-colonizing capabilities and has effectively been utilized to administer tumor-specific neoantigens (e.g., CT26-derived MHCIa, MHCIIa, MHCI/IIv), therapeutic cytokines (IL-2), and nanobodies, all of which elicit robust systemic immune responses [55,56,57]. Significantly, our investigation demonstrated that GPC1 antigen was expressed intracellularly within EcN, unlike Kitagawa’s method of surface antigen presentation in Bifidobacterium, hence providing enhanced stability of antigen retention and delivery. Although its potential has been established, no previous research has investigated EcN for oral antigen delivery in cancer vaccines, rendering our method a notable advancement in the field.

These data highlight the necessity for additional research into the molecular processes associated with antigen presentation and immune regulation by vaccinations delivered through mucosal routes. A comprehensive comprehension of these processes is essential for the advancement of more efficient mucosal vaccinations that not only hinder disease but also regulate the immune system effectively without causing excessive tolerance. These improvements will play a crucial role in the field of mucosal immunology, especially for vaccines that aim to target complicated antigens like tumor-associated antigens.

The genetically engineered constructs and transfected PANC02 cell lines developed in this study provide critical tools for assessing the antigen specificity of cytotoxic T cells in vitro. These tools are particularly valuable for elucidating the immune response mechanisms following vaccination with bacteria engineered to express GPC1. By using these GPC1-expressing PANC02 cells as a model, researchers can precisely monitor and analyze how cytotoxic T cells recognize and react to the GPC1 antigen presented by tumor cells, offering insights into the potential efficacy of GPC1-based immunotherapeutic strategies against pancreatic ductal adenocarcinoma.

The study created genetically engineered constructs and transfected PANC02 cell lines, which are essential tools for evaluating the antigen specificity of cytotoxic T cells in vitro. Researchers can utilize the GPC1-expressing PANC02 cells as a model to accurately observe and study how cytotoxic T cells identify and respond to the GPC1 antigen displayed by tumor cells. This provides valuable information on the effectiveness of GPC1-based immunotherapeutic approaches for treating pancreatic ductal adenocarcinoma.

The results of our study provide additional evidence that the introduction of GPC1 into PANC02 cells through transfection does not have a negative impact on their ability to multiply. This is supported by the similar rates of growth observed in both the GPC1-transfected cells and the control cells. This observation is significant since it indicates that the production of the GPC1 protein does not disrupt the cell cycle or cause cytotoxicity in these cells. The consistent growth features of the tumor cells guarantee that any detected anti-tumor immune response may be more accurately linked to the immune system’s interaction with the GPC1 antigen, rather than alterations in the fundamental development dynamics of the tumor cells.

This stability not only validates the appropriateness of GPC1 as a target for immune-based therapy, but also emphasizes the possibility of using GPC1-expressing vectors safely in additional preclinical and clinical investigations. These findings provide a basis for further research on the impact of GPC1-modified tumor cells on immune responses, which could potentially result in novel and efficient treatments for pancreatic cancer.

This study presents a novel method for cancer immunotherapy utilizing the probiotic *Escherichia coli* Nissle 1917 (EcN) as an oral delivery system for Glypican-1 (GPC1), a significant tumor-associated antigen in pancreatic cancer treatment. This research broadens vaccine delivery methods and leverages the inherent characteristics of probiotics to improve immunogenicity and patient compliance via non-invasive administration.

This study provides empirical evidence that genetically altered EcN may effectively deliver GPC1 to the immune system through mucosal pathways, triggering a robust immunological response marked by elevated systemic levels of IgG and the regulatory cytokine IL-10. These findings not only confirm the efficacy of microbial vectors in vaccine administration but also set a new standard in the field by demonstrating that oral delivery methods can produce immune modulation comparable to, or possibly exceeding, conventional invasive procedures.

The broader ramifications of our findings extend beyond pancreatic cancer, presenting feasible pathways for the creation of vaccines aimed at other diseases necessitating targeted immune activation. This technique employs a biocompatible and non-invasive delivery mechanism, ensuring optimal patient safety and tolerability, thereby establishing it as a paradigm for clinical translation. As the demand for more effective and minimally invasive vaccination methods increases, our findings provide a crucial contribution to the scientific discussion, illustrating that genetically modified probiotics like EcN can be utilized as powerful tools in vaccine delivery. The incorporation of this technology is poised to boost immunotherapy and revolutionize vaccine development and administration in future applications. This innovative methodology combines with the current demands and future directions of vaccine science, offering a scalable and effective framework for developing advanced immunotherapeutic tactics that are both powerful and beneficial to patient health. As we continue to improve and expand this technology, the potential for advancing and protecting human health through advanced vaccination delivery methods is becoming increasingly attainable.

## 5. Limitations and Future Perspectives

This study demonstrates the feasibility of *Escherichia coli* Nissle 1917 (EcN) as an oral vaccine platform for GPC1 antigen delivery; yet, several limitations must be considered for a balanced interpretation. Due to budgetary and logistical constraints, the study employed a relatively small sample size, which may have limited the statistical power necessary to detect subtle but biologically relevant differences, particularly in cytokine levels such as IL-2 and IFN-γ. The absence of significant differences in these markers, despite observed trends, underscores the need for larger cohorts and extended follow-up to capture the full immunological landscape and assess long-term durability.

Further, antibody isotyping was not performed, owing to resource limitations and focus on initial proof-of-concept endpoints. This restricts a complete understanding of the Th1 versus Th2 polarization of the immune response. Future studies should incorporate comprehensive isotyping of IgG subclasses (e.g., IgG1, IgG2a), alongside mucosal IgA quantification, enabling precise differentiation between systemic and mucosal immunity and clarifying the immune polarization elicited by EcN-based vaccination.

Additionally, the study design did not include in vivo tumor challenge experiments or survival analyses, which are critical for translating immunogenicity findings into clinically meaningful anti-tumor efficacy. Incorporating tumor inoculation models, combined with vaccination and survival tracking, will be essential to assess vaccine-mediated tumor control and immunological memory.

Another constraint was the focus on intracellular expression of GPC1 in EcN. Future research should explore surface display strategies and co-delivery with mucosal adjuvants, which may enhance antigen presentation and T cell priming. Similarly, detailed immunophenotyping of T cell subsets, particularly cytotoxic CD8^+^ T cells and regulatory T cells, will be necessary to fully characterize the cellular immune landscape. Lastly, microbiome profiling was beyond the scope of this study but represents a vital area to investigate, as interactions between EcN colonization and gut microbiota may significantly influence systemic immune responses.

Despite these limitations, this study provides a robust foundation and proof-of-concept for EcN-mediated oral cancer vaccines, opening avenues for optimized formulations, larger-scale validations, and preclinical efficacy studies.

## 6. Conclusions

This study introduces an innovative method for cancer immunotherapy utilizing genetically modified *Escherichia coli* Nissle 1917 (EcN) as an oral delivery mechanism for Glypican-1 (GPC1), a tumor-associated antigen. Our findings indicate that EcN serves as a safe and effective vehicle for antigen delivery, underscoring its potential as a non-invasive alternative to conventional vaccine platforms. The production of GPC1 in the gastrointestinal tract of mice, along with the observed immunological response, validates the potential of utilizing EcN to stimulate both mucosal and systemic immunity.

The robust immunological response induced by EcN was accompanied by a significant increase in the regulatory cytokine IL-10, indicating that the immune activation is predominantly regulatory rather than inflammatory. This indicates a necessity for more optimization of the vaccine, specifically to augment T cell activation and boost tumor-specific immunity. This discovery enhances the potential of microbial-based vaccinations, demonstrating that EcN may be pivotal in future cancer immunotherapy strategies. It establishes a robust basis for subsequent investigations aimed at enhancing this technique, potentially resulting in more accessible and patient-friendly cancer vaccines. The ongoing refinement of this method indicates a growing promise for revolutionizing cancer immunotherapy through probiotic-based platforms.

## Data Availability

The data supporting the results reported in this study are included in the article and its Supplementary Materials.

## Supplementary Materials

The following supporting information can be downloaded at: https://www.mdpi.com/article/10.3390/medicina61040633/s1, Figure S1: Western blot of GPC1-Flagellin expression in *Escherichia coli* Nissle 1917. Figure S2: Western blot of GPC1 expression in various cell lines. Figure S3: Flow cytometry analysis of GPC1 expression in PANC02 cells. Figure S4: Western blot of fecal samples detecting GPC1-FL protein post-immunization.

## References

[1] Stoffel E.M., Brand R.E., Goggins M. (2023). Pancreatic Cancer: Changing Epidemiology and New Approaches to Risk Assessment, Early Detection, and Prevention. Gastroenterology.

[2] Usami M., Kikuchi S., Takada K., Ono M., Sugama Y., Arihara Y., Hayasaka N., Nakamura H., Ikeda Y., Hirakawa M. (2020). FOXO3a Activation by HDAC Class IIa Inhibition Induces Cell Cycle Arrest in Pancreatic Cancer Cells. Pancreas.

[3] Warshaw A.L., Castillo C.F.-d. (1992). Pancreatic Carcinoma. N. Engl. J. Med..

[4] Fathi M., Ghafouri-Fard S., Abak A., Taheri M. (2021). Emerging roles of miRNAs in the development of pancreatic cancer. Biomed. Pharmacother. = Biomed. Pharmacother..

[5] Sohal D.P., Mangu P.B., Khorana A.A., Shah M.A., Philip P.A., O’reilly E.M., Uronis H.E., Ramanathan R.K., Crane C.H., Engebretson A. (2016). Metastatic Pancreatic Cancer: American Society of Clinical Oncology Clinical Practice Guideline. JCO.

[6] Jamali E., Safarzadeh A., Hussen B.M., Liehr T., Ghafouri-Fard S., Taheri M. (2023). Single cell RNA-seq analysis with a systems biology approach to recognize important differentially expressed genes in pancreatic ductal adenocarcinoma compared to adjacent non-cancerous samples by targeting pancreatic endothelial cells. Pathol. Res. Pract..

[7] Perez K., Clancy T.E., Mancias J.D., Rosenthal M.H., Wolpin B.M. (2017). When, What, and Why of Perioperative Treatment of Potentially Curable Pancreatic Adenocarcinoma. JCO.

[8] Muthukutty P., Woo H.Y., Ragothaman M., Yoo S.Y. (2023). Recent Advances in Cancer Immunotherapy Delivery Modalities. Pharmaceutics.

[9] Muthukutty P., Yoo S.Y. (2023). Oncolytic Virus Engineering and Utilizations: Cancer Immunotherapy Perspective. Viruses.

[10] Foley K., Kim V., Jaffee E., Zheng L. (2016). Current progress in immunotherapy for pancreatic cancer. Cancer Lett..

[11] Schlom J. (2012). Therapeutic Cancer Vaccines: Current Status and Moving Forward. JNCI J. Natl. Cancer Inst..

[12] Wang R.F., Robbins P.F., Kawakami Y., Kang X.Q., Rosenberg S.A. (1995). Identification of a gene encoding a melanoma tumor antigen recognized by HLA-A31-restricted tumor-infiltrating lymphocytes. J. Exp. Med..

[13] Wang R.F., Appella E., Kawakami Y., Kang X., Rosenberg S.A. (1996). Identification of TRP-2 as a human tumor antigen recognized by cytotoxic T lymphocytes. J. Exp. Med..

[14] Yarchoan M., Johnson B.A., Lutz E.R., Laheru D.A., Jaffee E.M. (2017). Targeting neoantigens to augment antitumour immunity. Nat. Rev. Cancer.

[15] Iqbal N., Iqbal N. (2014). Human Epidermal Growth Factor Receptor 2 (HER2) in Cancers: Overexpression and Therapeutic Implications. Mol. Biol. Int..

[16] Bakker A.B., Schreurs M.W., de Boer A.J., Kawakami Y., A Rosenberg S., Adema G.J., Figdor C.G. (1994). Melanocyte lineage-specific antigen gp100 is recognized by melanoma-derived tumor-infiltrating lymphocytes. J. Exp. Med..

[17] Vennegoor C., Hageman P., Vannouhuijs H., Ruiter D., Calafat J., Ringens P., Rumke P. (1988). A monoclonal antibody specific for cells of the melanocyte lineage. Am. J. Pathol..

[18] Busato D., Mossenta M., Dal Bo M., Macor P., Toffoli G. (2022). The Proteoglycan Glypican-1 as a Possible Candidate for Innovative Targeted Therapeutic Strategies for Pancreatic Ductal Adenocarcinoma. Int. J. Mol. Sci..

[19] Tsujii S., Serada S., Fujimoto M., Uemura S., Namikawa T., Nomura T., Naka T. (2021). Glypican-1 Is a Novel Target for Stroma and Tumor Cell Dual-Targeting Antibody-Drug Conjugates in Pancreatic Cancer. Mol. Cancer Ther..

[20] Aikawa T., Whipple C.A., Lopez M.E., Gunn J., Young A., Lander A.D., Korc M. (2008). Glypican-1 modulates the angiogenic and metastatic potential of human and mouse cancer cells. J. Clin. Investig..

[21] Kleeff J., Ishiwata T., Kumbasar A., Friess H., Büchler M.W., Lander A.D., Korc M. (1998). The cell-surface heparan sulfate proteoglycan glypican-1 regulates growth factor action in pancreatic carcinoma cells and is overexpressed in human pancreatic cancer. J. Clin. Investig..

[22] Tanaka M., Ishikawa S., Ushiku T., Morikawa T., Isagawa T., Yamagishi M., Yamamoto H., Katoh H., Takeshita K., Arita J. (2017). EVI1 modulates oncogenic role of GPC1 in pancreatic carcinogenesis. Oncotarget.

[23] Li J., Kleeff J., Kayed H., Felix K., Penzel R., Büchler M.W., Korc M., Friess H. (2004). Glypican-1 antisense transfection modulates TGF-β-dependent signaling in Colo-357 pancreatic cancer cells. Biochem. Biophys. Res. Commun..

[24] Sayad A., Najafi S., Hussen B.M., Jamali E., Taheri M., Ghafouri-Fard S. (2022). The role of circular RNAs in pancreatic cancer: New players in tumorigenesis and potential biomarkers. Pathol.-Res. Pract..

[25] Gambirasi M., Safa A., Vruzhaj I., Giacomin A., Sartor F., Toffoli G. (2024). Oral Administration of Cancer Vaccines: Challenges and Future Perspectives. Vaccines.

[26] Ueki H., Kitagawa K., Kato M., Yanase S., Okamura Y., Bando Y., Hara T., Terakawa T., Furukawa J., Nakano Y. (2023). An oral cancer vaccine using Bifidobacterium vector augments combination of anti-PD-1 and anti-CTLA-4 antibodies in mouse renal cell carcinoma model. Sci. Rep..

[27] Kitagawa K., Tatsumi M., Kato M., Komai S., Doi H., Hashii Y., Katayama T., Fujisawa M., Shirakawa T. (2021). An oral cancer vaccine using a Bifidobacterium vector suppresses tumor growth in a syngeneic mouse bladder cancer model. Mol. Ther.-Oncolytics.

[28] Critchley-Thorne R.J., Stagg A.J., Vassaux G. (2006). Recombinant *Escherichia coli* Expressing Invasin Targets the Peyer’s Patches: The Basis for a Bacterial Formulation for Oral Vaccination. Mol. Ther..

[29] Critchley R.J., Jezzard S., Radford K.J., Goussard S., Lemoine N.R., Grillot-Courvalin C., Vassaux G. (2004). Potential therapeutic applications of recombinant, invasive *E. coli*. Gene Ther..

[30] Hu P.Q., Tuma-Warrino R.J., Bryan M.A., Mitchell K.G., Higgins D.E., Watkins S.C., Salter R.D. (2004). *Escherichia coli* Expressing Recombinant Antigen and Listeriolysin O Stimulate Class I-Restricted CD8+ T Cells following Uptake by Human APC1. J. Immunol..

[31] Radford K.J., E Higgins D., Pasquini S., Cheadle E.J., Carta L., Jackson A.M., Lemoine N.R., Vassaux G. (2002). A recombinant *E. coli* vaccine to promote MHC class I-dependent antigen presentation: Application to cancer immunotherapy. Gene Ther..

[32] Remer K.A., Bartrow M., Roeger B., Moll H., Sonnenborn U., Oelschlaeger T.A. (2009). Split immune response after oral vaccination of mice with recombinant *Escherichia coli* Nissle 1917 expressing fimbrial adhesin K88. Int. J. Med. Microbiol..

[33] Zyrek A.A., Cichon C., Helms S., Enders C., Sonnenborn U., Schmidt M.A. (2007). Molecular mechanisms underlying the probiotic effects of *Escherichia coli* Nissle 1917 involve ZO-2 and PKCzeta redistribution resulting in tight junction and epithelial barrier repair. Cell. Microbiol..

[34] Schultz M. (2008). Clinical use of *E. coli* Nissle 1917 in inflammatory bowel disease. Inflamm. Bowel Dis..

[35] Sarnelli G., Del Re A., Pesce M., Lu J., Esposito G., Sanseverino W., Corpetti C., Franzin S.B., Seguella L., Palenca I. (2023). Oral Immunization with *Escherichia coli* Nissle 1917 Expressing SARS-CoV-2 Spike Protein Induces Mucosal and Systemic Antibody Responses in Mice. Biomolecules.

[36] Lu H., Niu F., Liu F., Gao J., Sun Y., Zhao X. (2017). Elevated glypican-1 expression is associated with an unfavorable prognosis in pancreatic ductal adenocarcinoma. Cancer Med..

[37] Altenhoefer A., Oswald S., Sonnenborn U., Enders C., Schulze J., Hacker J., Oelschlaeger T.A. (2004). The probiotic *Escherichia coli* strain Nissle 1917 interferes with invasion of human intestinal epithelial cells by different enteroinvasive bacterial pathogens. FEMS Immunol. Med. Microbiol..

[38] Westendorf A.M., Gunzer F., Deppenmeier S., Tapadar D., Hunger J.K., Schmidt M.A., Buer J., Bruder D. (2005). Intestinal immunity of *Escherichia coli* NISSLE 1917, a safe carrier for therapeutic molecules. FEMS Immunol. Med. Microbiol..

[39] Dubbert S., Klinkert B., Schimiczek M., Wassenaar T.M., Bünau R.V. (2020). No Genotoxicity Is Detectable for *Escherichia coli* Strain Nissle 1917 by Standard In Vitro and In Vivo Tests. Eur. J. Microbiol. Immunol..

[40] Smith H.W. (1975). Survival of orally administered *E. coli* K 12 in alimentary tract of man. Nature.

[41] Schultz M., Watzl S., Oelschlaeger T.A., Rath H.C., Göttl C., Lehn N., Schölmerich J., Linde H.-J. (2005). Green fluorescent protein for detection of the probiotic microorganism *Escherichia coli* strain Nissle 1917 (EcN) in vivo. J. Microbiol. Methods.

[42] Schultz M., Strauch U.G., Linde H.J., Watzl S., Obermeier F., Göttl C., Dunger N., Grunwald N., Schölmerich J., Rath H.C. (2004). Preventive Effects of *Escherichia coli* Strain Nissle 1917 on Acute and Chronic Intestinal Inflammation in Two Different Murine Models of Colitis. Clin. Vaccine Immunol..

[43] Gunzer F., Hennig-Pauka I., Waldmann K.-H., Sandhoff R., Gröne H.-J., Kreipe H.-H., Matussek A., Mengel M. (2002). Gnotobiotic Piglets Develop Thrombotic Microangiopathy After Oral Infection With Enterohemorrhagic *Escherichia coli*. Am. J. Clin. Pathol..

[44] Brandtzaeg P., Pabst R. (2004). Let’s go mucosal: Communication on slippery ground. Trends Immunol..

[45] Brandtzaeg P., Johansen F.E. (2005). Mucosal B cells: Phenotypic characteristics, transcriptional regulation, and homing properties. Immunol. Rev..

[46] Botía-Sánchez M., Alarcón-Riquelme M.E., Galicia G. (2021). B Cells and Microbiota in Autoimmunity. IJMS.

[47] Luckheeram R.V., Zhou R., Verma A.D., Xia B. (2012). CD4^+^T cells: Differentiation and functions. Clin. Dev. Immunol..

[48] Gonnella P.A., Chen Y., Inobe J., Komagata Y., Quartulli M., Weiner H.L. (1998). In situ immune response in gut-associated lymphoid tissue (GALT) following oral antigen in TCR-transgenic mice. J. Immunol..

[49] Qiao N., Du G., Zhong X., Sun X. (2021). Recombinant lactic acid bacteria as promising vectors for mucosal vaccination. Exploration.

[50] Kitagawa K., Oda T., Saito H., Araki A., Gonoi R., Shigemura K., Hashii Y., Katayama T., Fujisawa M., Shirakawa T. (2017). Development of oral cancer vaccine using recombinant Bifidobacterium displaying Wilms’ tumor 1 protein. Cancer Immunol. Immunother..

[51] Kitagawa K., Gonoi R., Tatsumi M., Kadowaki M., Katayama T., Hashii Y., Shirakawa T. (2019). Preclinical Development of a WT1 Oral Cancer Vaccine Using a Bacterial Vector to Treat Castration-Resistant Prostate Cancer. Mol. Cancer Ther..

[52] He L., Yang H., Tang J., Liu Z., Chen Y., Lu B., He H., Tang S., Sun Y., Liu F. (2019). Intestinal probiotics *E. coli* Nissle 1917 as a targeted vehicle for delivery of p53 and Tum-5 to solid tumors for cancer therapy. J. Biol. Eng..

[53] Kullen M.J., Klaenhammer T.R. (2000). Genetic modification of intestinal lactobacilli and bifidobacteria. Curr. Issues Mol. Biol..

[54] Ou B., Yang Y., Tham W.L., Chen L., Guo J., Zhu G. (2016). Genetic engineering of probiotic *Escherichia coli* Nissle 1917 for clinical application. Appl. Microbiol. Biotechnol..

[55] Redenti A., Im J., Redenti B., Li F., Rouanne M., Sheng Z., Sun W., Gurbatri C.R., Huang S., Komaranchath M. (2024). Probiotic neoantigen delivery vectors for precision cancer immunotherapy. Nature.

[56] Canale F.P., Basso C., Antonini G., Perotti M., Li N., Sokolovska A., Neumann J., James M.J., Geiger S., Jin W. (2021). Metabolic modulation of tumours with engineered bacteria for immunotherapy. Nature.

[57] Gurbatri C.R., Lia I., Vincent R., Coker C., Castro S., Treuting P.M., Hinchliffe T.E., Arpaia N., Danino T. (2020). Engineered probiotics for local tumor delivery of checkpoint blockade nanobodies. Sci. Transl. Med..

