# Supplementary material for: Gut Microbiota-Based Immunotherapy: Engineered Escherichia coli Nissle 1917 for Oral Delivery of Glypican-1 in Pancreatic Cancer
Source: Medicina (Kaunas). 2025 Mar 30;61(4):633. doi: 10.3390/medicina61040633 (PMC12028767; doi:10.3390/medicina61040633)
Supplement: Supplementary file 1 [file medicina-61-00633-s001.zip › medicina-3553977-supplementary.pdf]

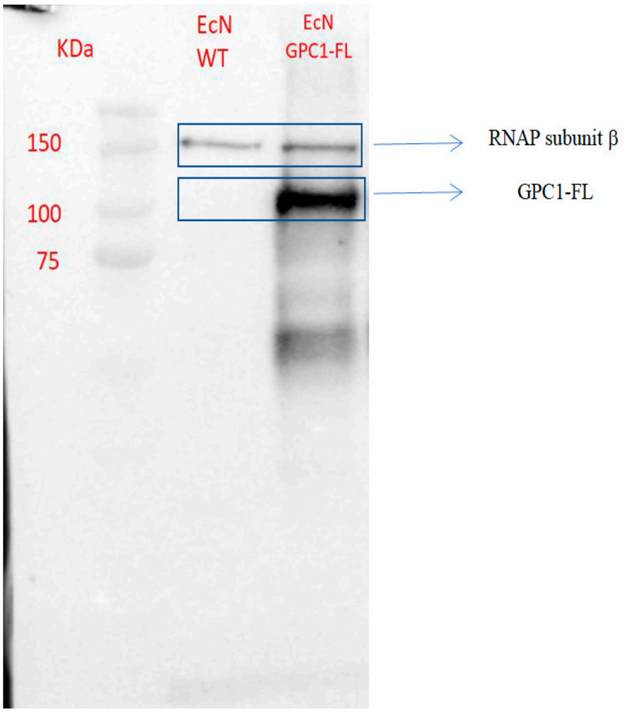

Supplementary Figure S1: Western blot analysis of GPC1-Flagellin (GPC1-FL) fusion protein expression in Escherichia coli Nissle 1917 (EcN). The protein ladder is shown on the left, with molecular weights labeled in kilodaltons (kDa). Lane 1 contains EcN wild-type (WT), showing the absence of the GPC1-FL protein. Lane 2 contains EcN transformed with the GPC1-FL construct, demonstrating the expression of GPC1-FL as a prominent band at approximately 100 kDa, corresponding to its expected molecular weight. A band corresponding to the RNA polymerase (RNAP) β-subunit (~150 kDa) is also visible as a loading control. This confirms the successful expression of the GPC1-Flagellin fusion protein in the transformed EcN strain.

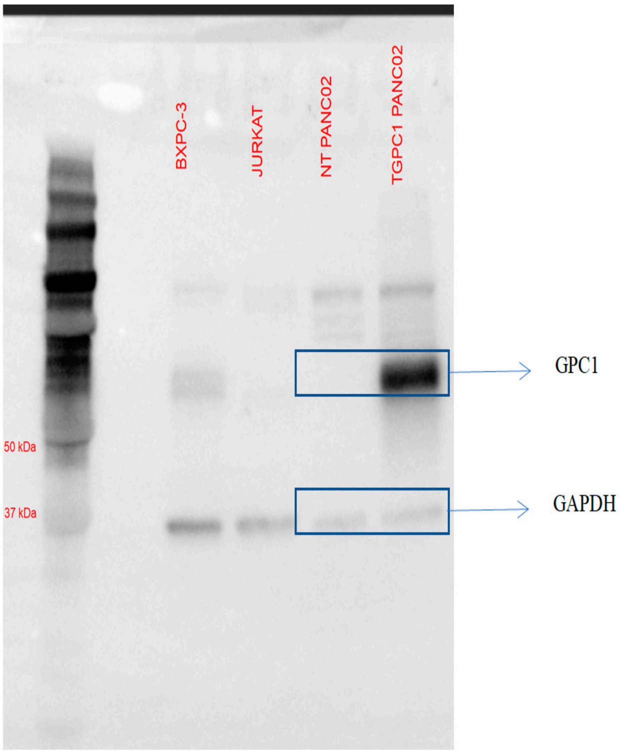

Supplementary Figure S2: Western blot analysis of GPC1 expression across various cell lines. The protein ladder is shown in Lane 1, with molecular weights labeled in kilodaltons (kDa). Lane 2 represents BXPC-3 cells, Lane 3 shows JURKAT cells, and Lane 4 displays wild-type (WT) PANC02 cells, all of which lack a detectable GPC1 band, indicating no expression of GPC1 in these cell lines. In contrast, Lane 5 represents transfected PANC02 cells (T-GPC1), showing a prominent band at the expected molecular weight for GPC1, confirming successful transfection and expression of the GPC1 gene. GAPDH is used as a loading control and is consistently detected across all lane

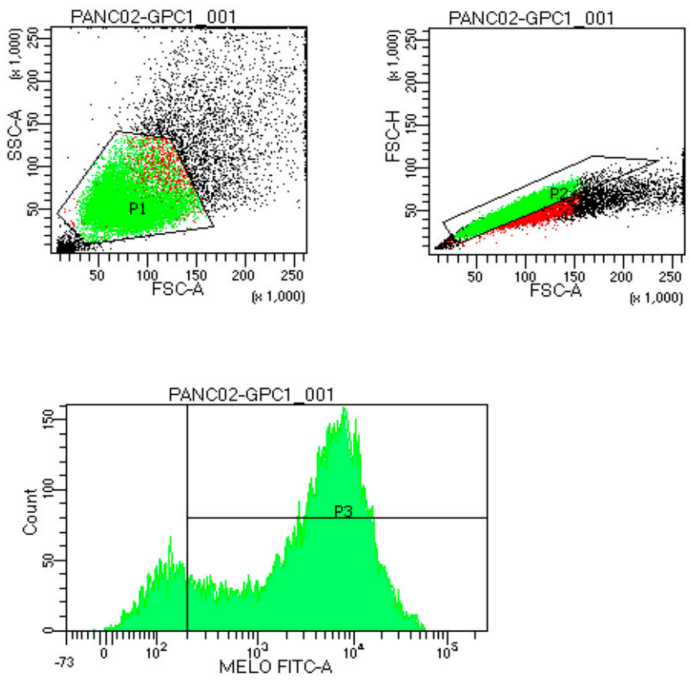

Supplementary Figure S3: Flow cytometry gating strategy for analyzing GPC1 expression in PANC02 cells. The first panel illustrates the initial gating to isolate PANC02 cells. The second panel shows gating for single cells, ensuring accurate analysis by excluding doublets or clusters. The third panel identifies PANC02 cells expressing GPC1, with 86.1% of the isolated cells positively expressing GPC1, confirming successful genetic modification and expression.

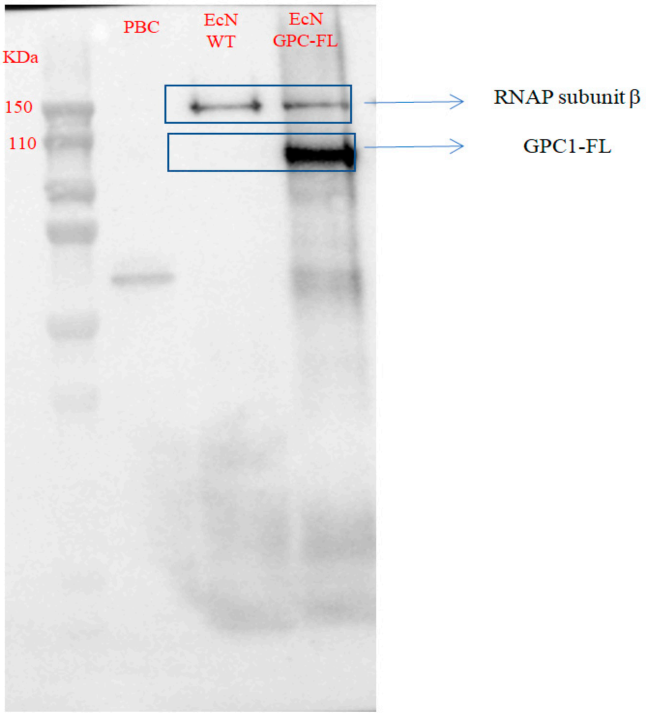

Supplementary Figure S4: Western blot analysis of fecal samples to detect GPC1-FL protein following oral immunization with genetically modified Escherichia coli Nissle (EcN) expressing GPC1-FL. Lane 1 shows the molecular weight marker. Lane 2 contains a sample labeled as PBC (negative control). Lane 3 represents stool samples from mice administered wild-type EcN (EcN WT), showing no detectable GPC1-FL protein. Lane 4 contains stool samples from mice vaccinated with EcN GPC-FL, displaying a distinct band corresponding to the GPC1-FL protein (~110 kDa). RNAP subunit β (~150 kDa) serves as a loading control and is consistently detected across all lanes. The results confirm successful expression of GPC1-FL in the EcN GPC-FL vaccinated group, with no expression observed in the control groups.
